# A Transverse Carpal Muscle Causing Carpal Tunnel Syndrome

**DOI:** 10.7759/cureus.7275

**Published:** 2020-03-15

**Authors:** Konstantinos Natsis, Maria Piagkou, Georgios Koimtzis, Aristeidis H Zibis

**Affiliations:** 1 Anatomy and Surgical Anatomy, Aristotle University of Thessaloniki, Thessaloniki, GRC; 2 Anatomy and Surgical Anatomy, National and Kapodistrian University of Athens, Athens, GRC; 3 General Surgery and Critical Care, Stepping Hill Hospital, Stockport, GBR; 4 Anatomy, School of Health Sciences, University of Thessaly, Larissa, GRC

**Keywords:** anomalous muscle, carpal tunnel syndrome, upper extremity, accessory muscle, compression, neuropathy, accessory innervation

## Abstract

Accessory and atypical muscles of the upper limb are common, whereas symptomatic variations presenting with carpal tunnel syndrome (CTS) are rare. A rare unilateral accessory transverse carpal muscle located palmar to the transverse carpal ligament is described. The accessory muscle, associated with CTS clinical manifestations in a 38-year-old Greek male worker, can be quite problematic during CTS operation. The current case emphasizes the importance of meticulous knowledge of the variable anatomy of the carpal tunnel (CT) area, since the accessory muscles may be accompanied by ectopic motor branches and a high risk of iatrogenic injury. Recognition and careful evaluation of accessory muscles may enhance a surgeon’s ability to carry out a safer and successful CT approach.

## Introduction

Although atypical and/or accessory muscles of the upper limb are common, their clinical manifestations causing carpal tunnel syndrome (CTS) are rare. CTS is a chronic peripheral compression neuropathy characterized by hand discomfort, swelling in the palmar surface of the wrist, pain and numbness in median nerve (MN) distribution, and thenar muscle weakness or atrophy. Clinical manifestations may be due to the increased pressure causing edema and synovial hypertrophy [1].

The existence of anomalous or variant muscles (palmaris longus [PL] variants), particularly the inverted or profundus form, the proximal origin of lumbrical muscles, the accessory belly of flexor digitorum superficialis and the transverse carpal muscle (TCM) may cause CTS symptoms, as well as bone fragments or tumors (lipomas and ganglionic cysts) [2-6]. The CTS occurs in 3.8% of the general population, with a higher prevalence in females and a bilateral appearance in 8-50% [7].

Taking into consideration that the anatomic variability during surgery enhances the surgeon’s ability to carry out a safe and successful approach to the carpal tunnel (CT), a rare unilateral accessory TCM located anterior to the transverse carpal ligament (TCL) associated with CTS is described, emphasizing to its clinical and surgical implications. Since CTS is a very common neuropathy, usually treated operatively, the surgeons must be prepared to overcome such muscular alterations in this region taking into consideration possible anatomical variants. The current report underlines the importance of meticulous knowledge of CT typical and variable anatomy, since the accessory muscles may be accompanied by ectopic motor branches and a high risk of iatrogenic injury.

## Case presentation

**Figure 1:**
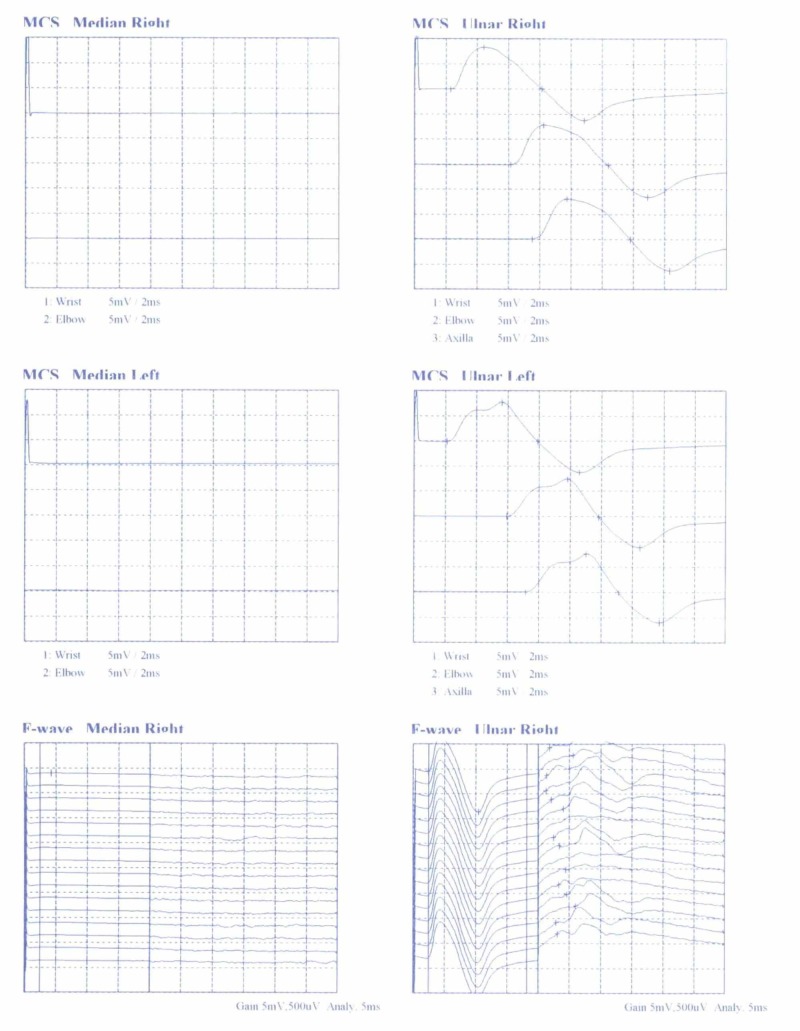
The nerve conduction study (NCS) showing median nerve neuroapraxia MCS - motor conduction study

**Figure 2:**
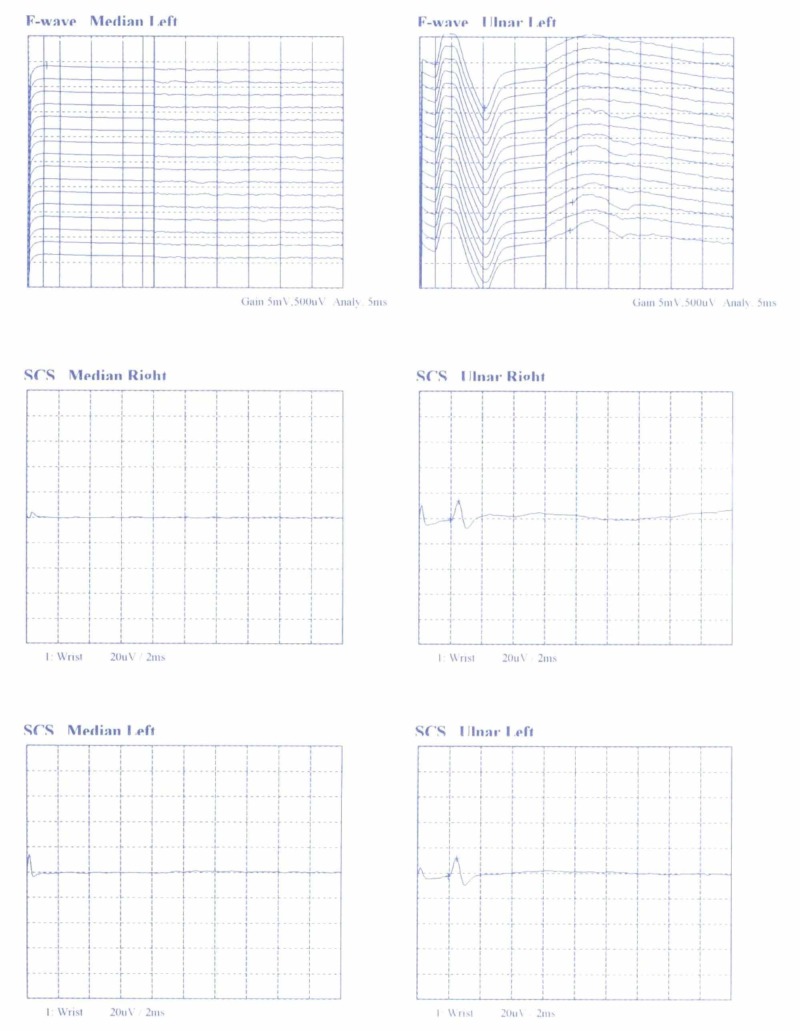
The nerve conduction study (NCS) showing median nerve neuroapraxia SCS - sensory conduction study

**Figure 3:**
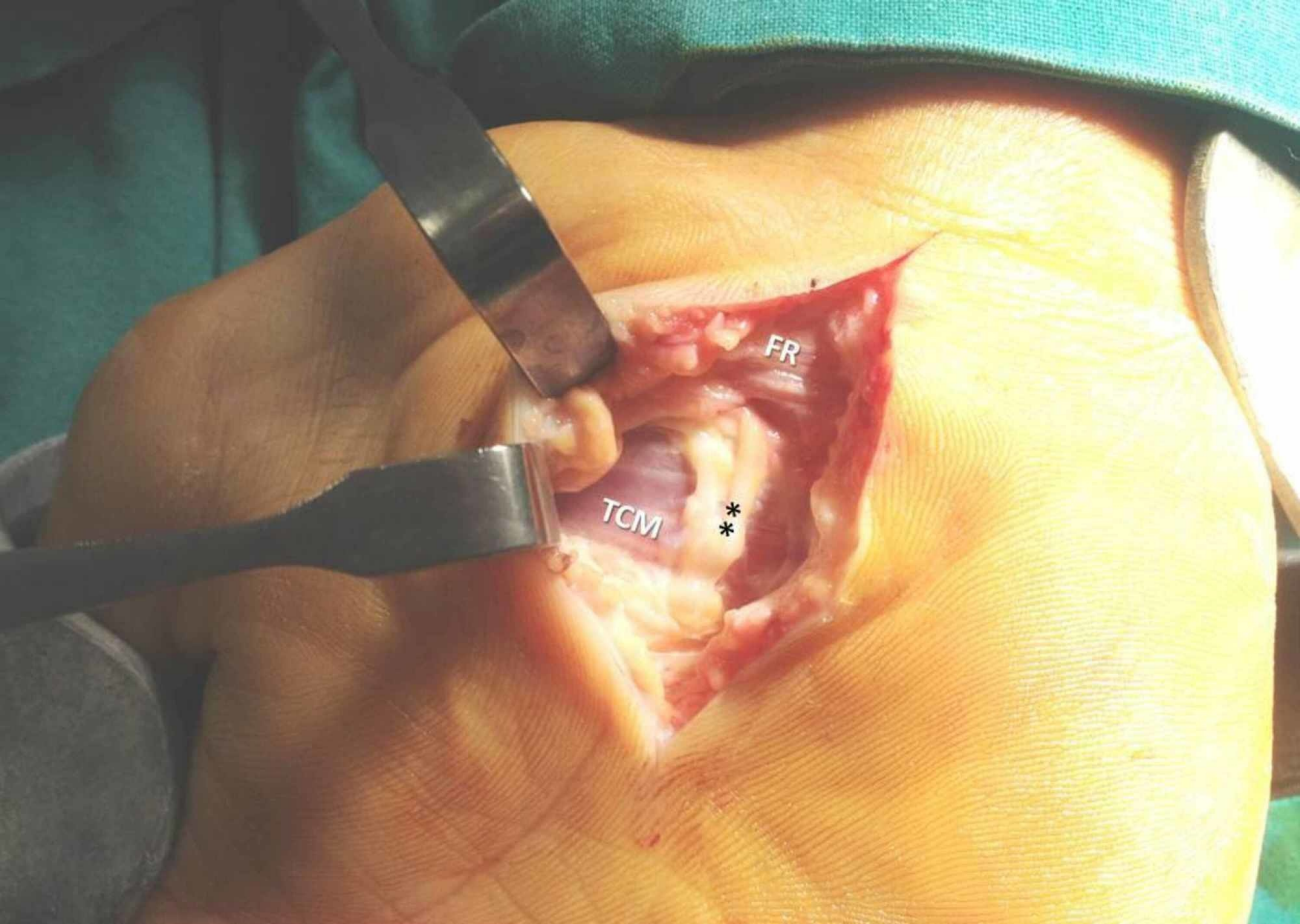
The accessory transverse carpal muscle (TCM) ** palmar cutaneous branch of the median nerve, FR- flexor retinaculum

**Figure 4:**
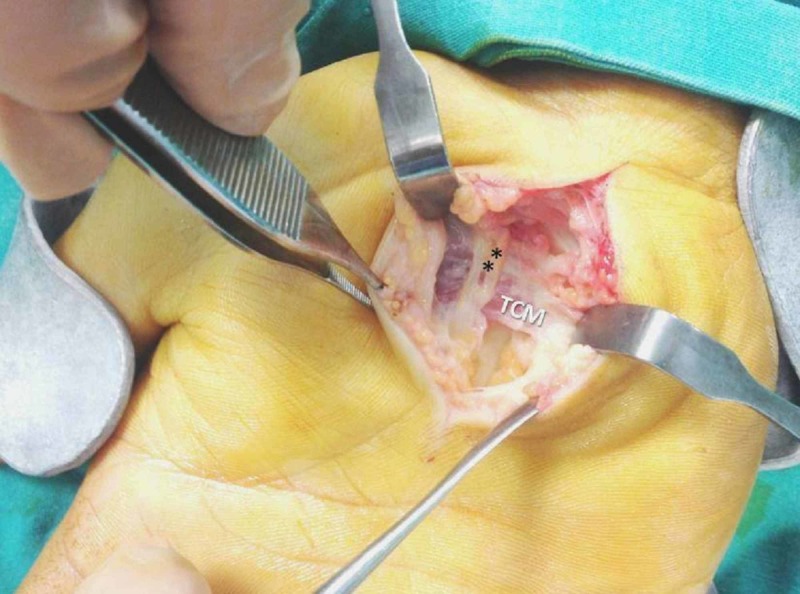
The accessory transverse carpal muscle (TCM) ** palmar cutaneous branch of the median nerve

A 38-year-old right-handed Greek male worker was presented with the typical signs and symptoms of CTS on his right hand. The patient referred to pain, paresthesia, decreased sensation, and weakness. The nerve conduction study (NCS) showed MN compression at the CT with sensory and motor conduction delay (Figures 1, 2). Following an open approach, after a longitudinal incision in the line of the third web space, skin and subcutaneous fat were incised, and the volar carpal ligament (superﬁcial layer of the deep antebrachial fascia) reached. After ligament’s incision, a transversely oriented muscle (dimensions 2.2 x 4.3 cm) overlapping the TCL and the flexor retinaculum was detected and named TCM. Its muscle bundles were oriented transversely, extending from thenar to hypothenar eminences, while the MN palmar branch overlapped TCM surface (Figures 3, 4). The TCM occurred only in the right wrist had no bony attachments. It was carefully incised ulnar to the MN and retracted proximally, while the TCL was incised, leading to MN decompression and immediate relief of the patient’s symptoms. The fibers’ width (5 mm) was measured with a ruler, as the distance between the proximal and distal border of muscle fibers on the line of flexor retinaculum incision. Due to the fact that the TCM was encountered intraoperatively, substantial dissection to demonstrate the exact origin, insertion, or innervation could not be undertaken. One month postoperatively, no functional deﬁcit was encountered and thenar muscle strength improved with time, while after two months, the contralateral hand showed signs of CTS, but during its surgical exploration, no similar muscle was found.

## Discussion

Although the aberrant hand muscles are common, they are rarely associated with compression neuropathies. Atypical hand muscle may originate from the thenar musculature, or it can be a hypertrophied palmaris brevis muscle (PBM), or a TCM in 11% of the cases [3, 8, 9]. The true identity of the muscles overlying or locating within the TCL is not definite. Mannerfelt and Hybbinette supported that the aberrant hand muscles were an aberrant PBM and a flexor pollicis brevis muscle (FPBM), but Shrewsbury et al. reported that the PBM never extended the ulnar margin of the palmar aponeurosis [5, 10]. Jonson and Shrewsbury noted that the FPBM and flexor digiti quinti blended into the TCL [11]. However, Green and Morgan noted that occasionally the muscles overlying the TCL had no apparent connection with the typical thenar or hypothenar muscles [12]. Ragoowansi et al. highlighted that these muscles belonged to a separate accessory muscle, the TCM [8].

In Gong et al. study, 51% of the Koreans had some muscle fibers over or within the TCL [13]. Hollevoet et al. found that muscle fiber in Caucasians had a width of 2-10 mm in 35% and 39% in both operated patients and cadaveric hands and a width of more than 10 mm in 11% and 15%, respectively [14]. Al-Qattan and Green and Morgan reported that 36% of the Middle Eastern patients and 28% of 1,400 CTS patients had hypertrophic muscle fibers over the TCL [12, 15].

Developmentally, the presence of the TCM may be the result of the aberrant migration of epiblastic cells from the "pronator quadratus" muscle that is transversely located proximal to the TCL [16].

In the current case, traction of the variant muscle did not cause any thumb movement or the hypothenar skin; thus, the muscle was not characterized as an accessory thenar muscle or a hypertrophied PBM. Cases of an anomalous PL with a muscular distal part have been associated with CT-like symptoms due to the increased pressure applied on the MN proximal to its insertion into the CT [17]. This correlation is well established in cases of a reversed PL with a muscular lower part [17]. TCM fibers may also be detected on the palmar surface of the flexor retinaculum and may have a role in CTS etiology [8, 9, 16]. Gong et al. supported that muscle fiber having a width up to 10 mm may represent ulnar extensions from thenar muscles or radial extensions from hypothenar muscles [13]. Occasionally, thenar and hypothenar muscles may blend across the flexor retinaculum. Sporadically, the detected muscle fibers may be derived from a hypertrophied palmaris brevis muscle or an accessory abductor digiti minimi longus crossing the flexor retinaculum in its proximal part [16]. Eversmann described a TCL of muscular appearance, possibly due to the thenar muscles’ enlargement and their ulnar extension [1]. In these cases, a high incidence of anomalous motor branches exists. Thus, the recognition of a muscular TCL should immediately alert the surgeon to proceed with extreme caution with ligament transaction, as the MN motor branch is at high risk of injury during the flexor retinaculum division. A wide TCM can be associated with an abnormal course of the MN motor branch, e.g., an ulnar-sided exit or a preligamentous course, as Hurwitz mentioned in his study on patients operated for CTS having an incidence of 9% [18]. Green and Morgan noted that special care should be taken to identify the motor branches when muscle fibers are encountered over the TCL because an atypical motor branch was detected in 93% of the cases [12]. They recommended not proceeding with TCL transection until the identification and protection of the motor branch ensue. Al-Qattan reported the presence of hypertrophic muscles over the TCL and their dissection over the radial side of the ligament, as the thenar motor branch is expected to be within the radial side of the hypertrophic muscle [15]. However, there have been reports, in which the motor branch originated from the medial side of the MN [13]. Thus, a careful layer-by-layer incision of the muscle is necessary.

Concerning the aberrant muscle participation in CTS symptomatology, in younger patients and manual workers, the possibility of MN compression by aberrant muscle fibers should be considered [2]. The contraction of the transverse muscle fibers may reduce CT diameter [9]. Anomalous muscles, when they are closely related to the nerves, may result in CTS or pressure neuritis [16]. In addition, patients with aberrant muscle fibers may present scar pain due to scar formation within the divided muscles.

Hand surgeons are accustomed to detecting TCM fibers running across the flexor retinaculum, contrariwise to inexperienced surgeons who may guide the incision too far to the radial side and lead to a more ulnar approach that could damage the ulnar artery and nerve [3]. Possible unexpected communications between MN and ulnar nerve should keep surgeons alert in the management of CTS [19]. TCM fibers may be difficult to manipulate during minimally invasive decompression [16]. No sufficient data exist concerning the role of wide TCM fibers in CTS causation. Thus no safe answer can be given if these muscles do show hypertrophy and thus produce CTS symptoms. The most plausible statement is that the anomalous muscle hypertrophy due to manual work is the causative factor for CTS. Ragoowansi et al. underlined that the occupation of the patient (carpenter), similarly to the current case (worker), had an impact on the detected muscle hypertrophy [8]. Nevertheless, CTS clinical manifestations in such cases cannot be explained by the ‘‘manual work’’ hypothesis alone. Similarly to our case, Tuncali et al. supported that the TCM fibers represented a separate muscle [16]. However, the surgical exposure is insufficient to determine the fibers’ origin running on the flexor retinaculum surface. To our knowledge, there are no reports of an isolated TCM found during dissection.

## Conclusions

The current case illustrates a variant TCM and emphasizes its importance during CT surgical exploration. The accessory TCM may be accompanied by a recurrent motor branch and the risk of its iatrogenic injury is evident. Future cadaveric studies to clarify the aberrant muscles’ attachments, blood supply, and innervation are essential to interpret variable entities.
